# Monthly Summary

**Published:** 1880-03

**Authors:** 


### MONTHLY SUMMARY.

*Duplicating Vulcanite Plates.*—I have fallen upon the following plan or modus operandi: When a plate is presented, it matters not how badly broken, so the pieces are all on hand, we unite the broken parts together with wax and model nicely, then mixing some plaster and pouring it into the lower section of the flask, press the plate into it, teeth looking downward, allowing the plaster to come up above the gums of the teeth, we slope it off towards the rim of the flask, then varnish and oil applied to the plaster, the inner side of plate being kept perfectly clean and free from plaster, varnish or oil. The second section of flask being now placed in position, the plaster is mixed and poured in and the flask carefully shaken until we are assured that every part of the plate is thoroughly covered. It is then allowed to harden perfectly. The vulcanizer is now filled half full of water and heat applied; when steam is raised, the flask is put therein. It is allowed to remain until it is thoroughly heated by a considerable amount of steam. The flask is then quickly removed, and rapidly, though carefully separated, and as soon as this is done, the rubber, which is now found in a semi-soft state, must be pulled hurriedly, though with some degree of care, out and away from the teeth and pins. It usually yields from them as readily as gutta percha base plates, and leaves the teeth intact, and pins clean and nice for packing. If it does not yield readily heat it again and remove at, before described. After this is accomplished, cut ways for excess of rubber; pack, vulcanize and finish in usual way. I have had such fine success by this method that I now never attempt to mend a plate in the ordinary way, but always duplicate. I find it requires less time, is decidedly more satisfactory, and usually for free charge, half price of a new plate. It is every whit as perfect as a new one. If a section of teeth is broken off I fit a new one in place, model with wax, imbed in plaster, and proceed as before described.

There are no doubt some to whom this method will be new, and if I have succeeded in giving them any new ideas on the subject of duplicating vulcanite plates, I will feel that I have been fully compensated for the efforts of this paper.—*Dr. W. R. Holmes, in Dent. Luminary.*

*Skin.—A Microscopical Study of its Inflammation.*—Dr. C. Heitzman, from his observations of inflamed skin, concludes: (1) In epithelium the first step of the inflammatory process consists in an increase of living both in and between the protoplasmic bodies, the former producing the coarse granulation of the epithelia, and the latter the thickening of the so-called horns in the cement substance. Any particle of living matter, either in the epithelia or between them, through continuous growth, may lead to a new formation of epithelial elements with the termination in hyperplasia of epithelium (psoriasis, squamous eczema, horny formations, etc.) (2) In connective tissues, the first manifestation of the inflammatory process is the dissolution of the basis substance and re-appearance of the protoplasmic condition by which process and the new formation of medullary elements the inflammatory infiltration is established. The sum total of the inflammatory elements which remain united with each other by means of delicate offshoots, represents an embryonal or medullary tissue. If the new formation of medullary elements be scanty, the resolution is accomplished by reformation of basis substance (erythema, erysipelas, etc.) If, on the contrary, the new formation of medullary elements be profuse, a new formation of connective tissue (hyperplasia) will result. (3) The plastic inflammation may be accompanied by the accumulation of a large amount of a serous or albuminous exudation in the epithelial layer (miliaria, udamina, herpes,) or in the connective tissue of the derma (urticaria. (4) Suppura-in the epithelial layer of the rete mucosum is produced by an accumulation of an albuminous or fibrinous exudation, by which a number of epithelia are destroyed, and by a new formation of pus corpuscles from the living matter of the epithelia themselves. (5) Suppuration in the connective tissue of the derma results from the breaking apart of the newly-formed medullary elements, which, being suspended in an albuminous or fibrinous exudation, now represent pus corpuscles. Pus is a product of the inflamed connective tissue itself, and always a result of destruction of this tissue.—*Boston Med. and Surg. Journal.*

---

*Uralium, a New Metal.*—As far back as 1869 the author discovered this metal in commercial platinum obtained from Russian ores. Next to silver it is the whitest metal known; its malleability is as great as that of the purest platinum, but its ductility is much greater, and it is almost as soft as lead. Its melting point lies near to that of platinum, and it is not volatile. Its specific gravity—20.25, and its molecular volume, like those of osmium, platinum and palladium, is 6.25. Its atomic weight has been found 187.25. In its chemical properties it is difficult to distinguish from platinum.—*A Guyard.*

*Excellent Glycerine Ointment.*—A very good preparation of glycerine to have always on hand, can be readily prepared by any apothecary or druggist: In two ounces of Sweet Oil of Almonds melt, by a slow heat, half an ounce of Spermaceti, and one drachm of White Wax. Then add one ounce of good Glycerine stirring until cold. When cold, scent it by stirring in well a little Oil of Roses. Keep in small jars or small wide-necked bottles. In hot weather keep closely corked, as it sometimes gets a little rancid if long exposed to warmth. Half or a fourth of the above quantities may be used. Every drug store should keep a jar of it, and recommend its use. It is excellent for softening the skin, for most injured skin surfaces that are not open sores; for chafed places, for moistening corns or callused feet or toes, and especially for chapped face, lips, or hands. When the hands are chapped or cracked, or roughened by cold, wash them clean with soap, and rub them well with this glycerine ointment, wiping it off enough to prevent soiling clothing. If this is done at night, the hands will be soft and in good condition in the morning, except when deeply cracked. It is very good to apply to the hands after "washing-day." This is an excellent preparation to use by those afflicted with the distressing trouble known as hæmorrhoids or piles.—*American Agriculturist.*

---

*Replanted Teeth.*—Miss K——, age eighteen years, called with left inferior first molar aching terribly; used various remedies to ease at once—no success. Extracted, removed partially devitalized nerve, cleaned thoroughly the two roots, wiped out with carbolic acid (Calvert's pure crystalized,) filled roots with gold moistened with the same; then filled the cavity, washed out the sockets of the roots with tepid water, and forced tooth into place, applied tincture of calendula to gums and parts every morning for three days. Very little pain was caused, and soreness all gone in three days. Same tooth a short time since was doing well after three years' use. No abscess formed.

Mr. S——, age twenty-five years, same as above, only first inferior bicuspid, used tincture calendula and tincture of iodine and aconite root, equal parts, alternately, for three days.—*Dent. Luminary.*

---

*Another Discoverer of Anesthesia.*—Anesthesia will soon have as many discoverers as Homer had birthplaces. In addition to the four already known to fame—Morton, Jackson, Wells and Long—a fifth claimant now appears in the person of a Dr. Wilhite, still living in South Carolina, a student of Dr. Long, to whom he imparted a knowledge of the subject. It is said that Wilhite's friends intend to push his claim.—*Pacific Med. and Surg. Journal.*

*The Physiology of Saliva.*—The action of saliva from the mouth in digestion has lately been studied by Herr Von der Velden. By extraction, with the pump, of numerous samples of gastric juice in various stages of digestive process it was demonstrated that in the first period after taking a meal (three-quarters to one, or even two hours) free hydrochloric acid did not appear; it was met with only later. Further, it was shown that only so long as the hydrochloric acid continues to be absent, is starch saccharified (iodide of potassium presents only a bright yellow color); whereas, after occurrence of the hydrochloric acid, the starch remains unaltered (blue coloration.) There is accordingly, a first stage of stomachic digestion, in which alone salivary action can take place; and a second, in which albumen digestion begins, or, at least, arrives at full intensity.—(*Med. Press and Circular.*

---

*Pathological Changes Observed in One Hundred Glaucomatous Eyes.*—Dr. Bailey (*Brit. Med. Jour.* Aug. 30, 1879,) gives the results of a microscopical study of the pathological changes observed in one hundred glaucomatous eyes. He found adhesion of the base of the iris to the periphery of the cornea; atrophy of the ciliary muscle, especially of its circular fibres; dilatation and thinning of the circulous arteriosus major; and in about half the cases similar changes in the long ciliary arteries. The canal of Schlemm was often diminished. The changes in the ciliary muscle and iris were sometimes confined to a part of the circle, and in some cases followed injury. These changes Dr. Bailey regards as proving that the atrophic changes referred to were the cause and not the consequence of the glaucomatous process. In the specimens from recent glaucoma there were adhesions at the base of the iris only.—*Boston Med. and Surg. Journal.*

---

*Chloral an antidote to Strychnine.*—Prof. Huseman, of Gottingen, has discovered that chloral hydrate is not only an antidote to strychnine, but to the mixture of strychnine bases, sold under the name of brucine. It has also a counteracting effect upon the opium alkaloid thebaia. But although chloral hydrate will stop the spasms caused by chloride of ammonium, the patient will die of paralysis of the respiratory centre, a result probably of the joint influence of both substances. In a certain degree the hydrate of chloral antagonizes picrotoxia, codeia, and calabaria. It is of little use in poisoning by baryta or carbolic acid. —*Exchange.*

*Replanting of Teeth.*—Dr. J. N. Prather, of San Francisco, Cal., writes: I notice in the August number, page 144, your paper, that "Dr. Thompson, of San Francisco, is now in London showing the profession there his new method of replanting teeth." That the minds of your readers may be further enlightened, I will state that replanting teeth is no new discovery, but has been practised more or less for ages. But the profesfion considers the replanting of teeth very impracticable, except in isolated cases. When a tooth is extracted the nerve is severed, and death to the tooth is inevitable; when returned to its former position, if properly anchored, it may adhere as strongly as ever to the surrounding parts, yet a tenderness will almost invariably be felt in mastication; they frequently act as irritants and ulcerate at the apex, thus becoming disagreeable and offensive, the crown turns dark, and it is thought they are more susceptible of decay.—*Med. Record.*

---

*The Audiphone.*—This is a new instrument for assisting the hearing of the moderately deaf. It consists of a flexible sheet of hard rubber with a handle attached, the whole resembling very much an ordinary fan. A string is attached in such a way that the rubber plate can be bent over towards the handle to a greater or less curve. The instrument is held in the hand and the top edge laid against the teeth, the convexity of the curved plate being out. The apparatus is quite ornamental, and is probably of assistance in some forms of deafness.—*Med. Record.*

---

*Ink on the Carpet.*—Ink freshly spilled upon the carpet should at once be taken up with soft paper or a slightly damp sponge, or even a damp cloth, care being exercised not to spread the spot. After all is taken up that can be, wet the sponge—after first washing it clean—in warm water, and thoroughly scrub the spot on the carpet. When no more can be washed out, wet the spot with a weak solution of Oxalic Acid, and after a few moments, wash off with cold water, and finally sponge with a weak Ammonia Water, to neutralize any of the acid that may remain in the carpet.—*American Agriculturist.*

---

*Japanese on Blood Letting.*—The Japanese do not countenance blood letting. They say the blood is too precious a fluid to be thus wasted. The copious drinking of hot water is one of their favorite prescriptions.

---

*Frequency of Bright's Disease.*—Nearly ten per cent. of the policy holders in a prominent life insurance company in this country are shown by statistics to die of Bright's disease.

